# Distinct etiology of chronic inflammation – implications on degenerative diseases and cancer therapy

**DOI:** 10.3389/fimmu.2024.1460302

**Published:** 2024-11-01

**Authors:** Krishna Rao Maddipati

**Affiliations:** Department of Pathology, Wayne State University, Detroit, MI, United States

**Keywords:** *unalamation*, cancer etiology, chronic inflammation, acute inflammation, prostaglandin E_2_, epoxy PUFA, endocannabinoids, chronic diseases

## Abstract

Acute inflammation is elicited by lipid and protein mediators in defense of the host following sterile or pathogen-driven injury. A common refrain is that chronic inflammation is a result of incomplete resolution of acute inflammation and behind the etiology of all chronic diseases, including cancer. However, mediators that participate in inflammation are also essential in homeostasis and developmental biology but without eliciting the clinical symptoms of inflammation. This non-inflammatory physiological activity of the so called ‘inflammatory’ mediators, apparently under the functional balance with anti-inflammatory mediators, is defined as *unalamation* (*un-ala-mation*). Inflammation in the absence of injury is a result of perturbance in *unalamation* due to a decrease in the anti-inflammatory mediators rather than an increase in the inflammatory mediators and leads to chronic inflammation. This concept on the etiology of chronic inflammation suggests that treatment of chronic diseases is better achieved by stimulating the endogenous anti-inflammatory mediators instead of inhibiting the ‘inflammatory’ mediator biosynthesis with Non-Steroidal Anti-Inflammatory Drugs (NSAIDs). Furthermore, both ‘inflammatory’ and anti-inflammatory mediators are present at higher concentrations in the tumor microenvironment compared to normal tissue environments. Since cancer is a proliferative disorder rather than a degenerative disease, it is proposed that heightened *unalamation*, rather than chronic inflammation, drives tumor growth. This understanding helps explain the inefficacy of NSAIDs as anticancer agents. Finally, inhibition of anti-inflammatory mediator biosynthesis in tumor tissues could imbalance *unalamation* toward local acute inflammation triggering an immune response to restore homeostasis and away from tumor growth.

## Introduction

Lipid mediators such as prostaglandin E_2_ and cytokines such as IL-6 participate both in acute and chronic inflammatory diseases. Non-Steroidal Anti-Inflammatory Drugs (NSAIDs), while highly effective in inhibiting the biosynthesis of the lipid mediators and acute inflammation, are ineffective to treat chronic illnesses such as rheumatoid arthritis, neurodegenerative, renal, cardiovascular, and myriad other diseases. Chronic inflammation is also implicated in sustaining tumor growth and metastasis. However, NSAIDs are also ineffective as anticancer drugs, yet the idea of chronic inflammation as the driver of tumor growth persists. A close examination of the implications of a recently proposed new paradigm called *unalamation (un-ala-mation)* (1) not only suggests a distinct etiology of chronic inflammation but also argues against the involvement of chronic inflammation in tumor growth. This novel understanding of the etiology of chronic inflammation offers radically different and revolutionary therapeutic development options.

## Essential historical perspective on inflammation

The clinical definition of inflammation, i.e., swelling, pain, redness, and heat, proposed during the first century of CE by Celsus survives to date with later addition of loss of organ function to the list by Galen (2). The first biological understanding of inflammation did not come until the late 19^th^ century when Ilya Metchnikoff proposed phagocytic theory of ‘physiological inflammation’ where the migration of phagocytes to digest the invading microbes results in the observed clinical symptoms (3). While prostaglandins were identified and their role in reproductive and vascular biology was ascertained by Bergstrom and Samuelsson in the 1950s and 60s, their role in inflammation was not apparent until they were first detected in inflammatory exudates by Willis in 1968 (4) and inhibition of their biosynthesis by non-steroidal anti-inflammatory drugs (NSAIDs) independently by Vane and Willis in 1971 (5, 6). Thus, the definition of inflammation has evolved from a description of the clinical symptoms at the organism level to an explanation by cell biology and finally to the molecular level by the identification of lipid mediators that instigate an inflammatory response. Presence of lipid mediators such as prostaglandin E_2_ (PGE_2_) and proteins such as IL-6 in inflammatory exudates and at the sites of inflammation have become biochemical hallmarks of inflammation.

## Acute and chronic inflammation

Inflammation is a physiological host defense mechanism against foreign substances, pathogens, and physical injury (7, 8). Whether the origin of the injury is sterile, or pathogen driven, inflammation helps restoration of systemic homeostasis following injury. Clinical manifestation of inflammation is characterized by increased blood flow and leakage of blood to the extravascular space to the site of injury resulting in swelling and redness. The febrile response to injury is mediated by enhanced PGE_2_ biosynthesis at the site of injury and signaled to the thermoregulatory region of the preoptic area of the hypothalamus (9). Activation of afferent nociceptors in the somatosensory neurons of the inflamed region by PGE_2_ results in pain perception (10). Lipid mediators such as prostaglandins and leukotrienes are the first responders to injury and initiate a cascade of molecular events that ultimately manifest in the clinical symptoms of inflammation (11). The very fact that non-steroidal anti-inflammatory drugs that target prostaglandin biosynthesis (but do not directly affect cytokine release) and can control inflammation, points to this hierarchy. Other inflammatory mediators such as cytokines quickly follow this initiation by prostaglandins and these mediators mutually enhance for the maximum effect of inflammation (i.e. to neutralize pathogens, clear the cell debris, etc.) before initiation of the resolution process to help return the system to homeostasis from injury (12). This is the model of acute inflammation resulting from the surge of lipid mediators at the site of injury and the basis of NSAID therapy. While collateral damage from severe acute inflammatory response is managed by NSAIDs and anti-cytokine therapy, ordered resolution of acute inflammation is actively assisted by Specialized Pro-resolving Mediators (SPMs), mostly derived from ω-3 polyunsaturated fatty acids (PUFA) (13, 14).

Chronic inflammation, on the other hand, need not necessarily manifest the clinical symptoms of acute inflammation overtly. In fact, cellular and molecular level analyses are essential to ascertain chronic inflammation when the symptoms are sub-clinical. While acute inflammation is resolved in hours to days and can effectively be controlled with NSAIDs, chronic inflammation can last for years to the life of an individual and NSAIDs are ineffective in the long run (15). Also, the etiology of acute inflammation in the form of injury is well defined than that of chronic inflammation, which is elusive. The mediators that sustain both acute and chronic inflammation, i.e., small molecules like prostaglandins and proteins like cytokines, are identical. However, it is the identity of the trigger that initiates the biosynthesis of lipid mediators and the subsequent release of cytokines that is elusive in almost all chronic inflammatory conditions.

## ‘Inflammatory mediator’ – inflammation disconnect

Interestingly, the presence (or even increase) of biochemical mediators that define inflammation is not unique to injury (16). Injury inevitably results in the spatiotemporal increase of the lipid mediators. Even exposure of tissues to exogenous mediators (the classic ‘rescue’ or ‘add back’ experiments where tissues are exposed *in vitro* or *in vivo* to the test compounds following the inhibition of their biosynthesis to prove their biological effect/role) also results in an inflammatory response. However, local increase of the ‘inflammatory’ mediators under normal physiological conditions does not necessarily result in the manifestation of inflammatory symptoms (17). In other words, increased lipid mediator biosynthesis and inflammation are coordinated *only following an injury* but a mere spatiotemporal increase in the mediators of ‘inflammation’ in the *absence* of injury does not result in inflammation. While normal renal, cardiovascular, neuronal, and intestinal physiology is replete with examples of high local levels of PGE_2_ without clinical symptoms of inflammation, this incongruity between the ‘inflammatory’ mediators and inflammation is best illustrated in reproductive biology.

Mammalian parturition is characterized by strong induction of cyclooxygenase-2 (COX-2) at term in the amnion (18, 19). This results in an increase of intra-amniotic concentrations of PGE_2_ and PGF_2_α, for example, in humans from virtually undetectable levels before the onset of labor to about 20 nM in the early stages and increases 2-3-fold further with labor progression (20–23). These high intra-amniotic concentrations of the ‘inflammatory’ lipid mediators are maintained until the delivery of the fetus. Amniotic cavity being a privileged space with highly restricted exchange of proteins and metabolites with the rest of the system, the concentrations of lipid mediators detected in the amniotic fluid are truly local (24). Biological activities of these lipid mediators are transduced through a set of tissue-specific degenerate receptors for PGE_2_ (EP1-4) and PGF_2_α (FP) (25, 26). Both EP3 and FP receptors are expressed in human myometrium at term and the binding constants for their respective ligands are 0.9 nM for EP3 and 3.4 nM for FP (27–29). In the injury/inflammation driven febrile response, activation of EP3 receptors by PGE_2_ is essential for raising the body temperature (fever) (30). Thus, both the biosynthesis and receptors are primed to exert maximal activity and to initiate an acute inflammatory response during parturition. However, in uncomplicated spontaneous labor, neither the mother nor the fetus experiences any clinical symptoms of inflammation (31).

Chorioamnionitis at term, on the other hand, is an inflammatory state characterized by maternal fever, maternal and fetal tachycardia, and neutrophil infiltration of the amniotic cavity commonly resulting from intra-amniotic infection (32, 33). Clinical symptoms of chorioamnionitis are also observed in the absence of microbial invasion of the amniotic cavity, i.e. sterile inflammation of unknown etiology (34). Sterile chorioamnionitis accounts for about 46% of all cases. Interestingly, neither sterile nor infection driven chorioamnionitis are associated with any further significant increase in the intra-amniotic levels of prostaglandins that are already at the receptor-saturating concentrations (35).

## Non-inflammatory physiology of ‘inflammatory’ mediators

**Figure 1:**
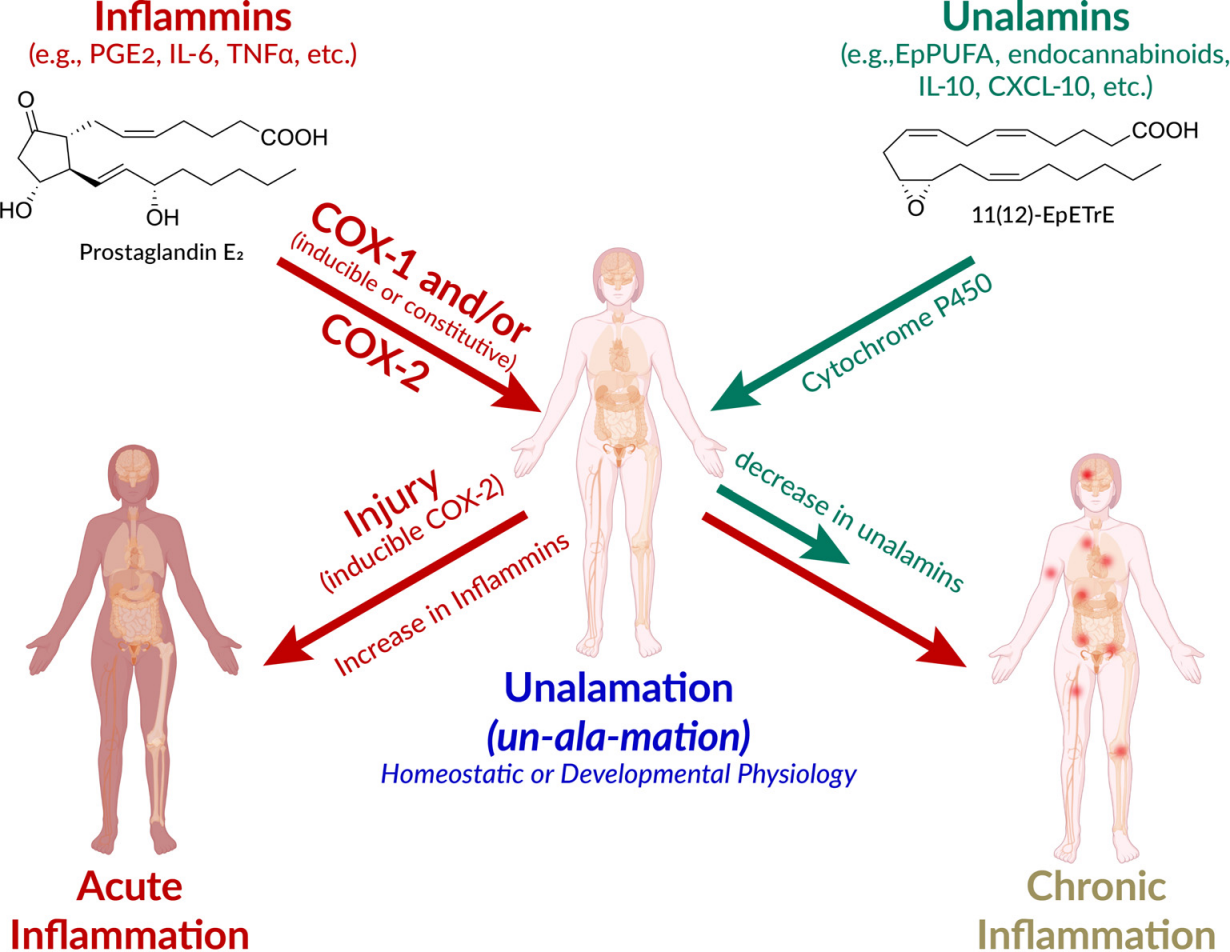
Schematic presentation of the pathways leading to acute and chronic inflammation and *unalamation*. Defensive immunological response to injury is acute inflammation mediated by lipid and protein mediators. However, the very same mediators also participate in homeostatic and developmental physiology. The designation of ‘inflammatory mediators’ for lipids such as prostaglandin E_2_ (PGE_2_) is therefore a misnomer. Instead, the generic term *inflammins* is coined to designate their propensity to elicit inflammation. These mediators elicit acute inflammation in the context of injury and inducible cyclooxygenase (COX)-2 is the initiator of this response. The anti-inflammatory mediators that repress the inflammatory traits of *inflammins* help maintain homeostasis are termed *unalamins.* Both *inflammins* and *unalamins* are small molecule (e.g. lipids such as PGE_2_, epoxy polyunsaturated fatty acids (EpPUFA), endocannabinoids, and/or protein mediators (e.g. cytokines). When *inflammins* are biosynthesized in response to injury, it is acute inflammation and serves a defensive function. On the other hand, *inflammins* routinely serve a normal developmental (e.g. in parturition, ovulation) and homeostatic (e.g. renal, cardiovascular, neuronal) physiological function under the pre-disposed control by *unalamins* and this is *Unalamation*. If the *unalamins* persistently fail to functionally regulate the *inflammins*, the result is chronic inflammation. (adapted and modified from *Front Immunol* 2020 Vol. 11 Pages 580117, Human body illustration from Biorender.com with permission).

Fatty acyl lipidomic analysis of human amniotic fluid at term shows that the lipid mediator profile also includes high levels of epoxy polyunsaturated fatty acids (EpPUFA) during parturition (**Table 1**) (24). While the anti-inflammatory properties of EpPUFA are known for a longtime (36, 37), little is known about the role of these cytochrome P450 pathway metabolites in parturition. Receptor biology of the EpPUFA is not yet elucidated but the effective concentration of these compounds to inhibit vascular inflammation (38, 39) and febrile response (40) as well as to maintain renal and neurovascular homeostasis (41) is in the range of 2 pM to 20 nM in model systems (42). Considering the presence of both pro- and anti-inflammatory lipids at high levels in the privileged locale of the amniotic cavity and the absence of any clinical symptoms of inflammation in uncomplicated spontaneous labor, one can surmise if the ‘inflammatory’ mediators serve a normal homeostatic purpose (both prostaglandins and EpPUFA are vasoactive, angiogenic, mitogenic, and regulate apoptosis) under the control of the anti-inflammatory EpPUFA. Interestingly, the lipidomic analysis also showed that clinical chorioamnionitis resulting from sterile inflammation is characterized by a decrease in the anti-inflammatory EpPUFA rather than an increase in prostaglandins (the so called ‘inflammatory’ mediators) levels (**Table 1**) (24, 35). This is not unique to lipid mediators either. This phenomenon is also replicated in the intra-amniotic cytokine concentrations as well, where the ‘inflammatory’ cytokines (IL-1β, IL-6, TNFα, etc.) remain the same but the ‘anti-inflammatory’ cytokines (e.g. IL-10, IL-13, CXCL-10, etc.) are lower in sterile inflammation (43). Many of the cytokines and chemokines are constitutively expressed under homeostatic conditions that are otherwise ‘inflammatory’ in the context of injury (11, 44). *In other words, inflammation can result not only from an increase in ‘inflammatory’ mediators but also a decrease in the ‘anti-inflammatory’ mediators that repress the inflammatory traits of lipid (and protein) mediators in their service of homeostatic physiology.* This observation led to the development of a new paradigm called *unalamation* (*un*-*ala*-*mation*)^1^ to explain the non-inflammatory physiology mediated by the very same mediators hitherto labeled as ‘inflammatory’ (**Figure 1**) (1). Resolution of inflammation is a pre-ordained active process mediated by SPMs that do not inhibit inflammation (13, 14). Because inflammation serves a defensive purpose (7), inhibition of inflammation prematurely is counterproductive to the organism. The presence of ‘anti-inflammatory’ lipid and protein mediators simultaneously with ‘inflammatory’ mediators in the same *in vivo* context is obviously inconsistent with the defensive function of inflammation. Hence, the role of endogenous ‘anti-inflammatory’ mediators is to sustain *unalamation* in coordination with the so called ‘inflammatory’ mediators.

**Table 1:** Concentrations of inflammatory and anti-inflammatory lipid mediators detected in human amniotic fluid at term in labor with or without inflammation.

Lipid Mediator	TLB [n=35]	TCC [n=18]	*p*
PGE_2_	65.2 (28.5 - 157.3) [34]	65.0 (23.6 - 115.4) [17]	0.86
19(R)-hydroxy PGE_2_	147.7 (88.4 - 243.2) [34]	138.8 (104.0 - 180.5) [18]	0.58
PGF_2_α	8.3 (0.0 - 24.6) [18]	9.3 (0.0 - 29.0) [10]	0.93
9(10)-EpOME	217.1 (172.2 - 384.9) [35]	113.1 (74.1 - 179.0) [18]	2.00 x 10^-4^
12(13)-EpOME	150.1 (106.5 - 212.7) [35]	59.9 (49.2 - 101.2) [18]	1.06 x 10^-4^
8(9)-EpETrE	39.3 (28.2 - 59.9) [34]	15.8 (10.0 - 22.6) [18]	1.27 x 10^-4^
11(12)-EpETrE	200.3 (177.7 - 307.6) [35]	79.2 (56.9 - 129.3) [18]	9.59 x 10^-5^
14(15)-EpETrE	109.6 (83.7 - 162.9) [35]	50.2 (24.0 - 71.6) [18]	1.67 x 10^-5^
8(9)-EpETE	2.9 (1.8 - 3.9) [30]	0.0 (0.0 - 0.0) [1]	2.21 x 10^-6^
11(12)-EpETE	14.6 (11.1 - 19.4) [35]	2.2 (0.4 - 3.8) [13]	5.00 x 10^-7^
17(18)-EpETE	5.9 (3.9 - 8.8) [35]	0.0 (0.0 - 1.2) [6]	3.10 x 10^-8^
16(17)-EpDPE	9.6 (7.4 - 14.5) [34]	3.0 (2.0 - 4.2) [16]	1.01 x 10^-5^
19(20)-EpDPE	13.4 (8.6 - 18.9) [35]	3.0 (2.6 - 5.0) [18]	3.93 x 10^-7^

TLB, spontaneous labor at term; TCC, clinical chorioamnionitis at term. Data (nM) are median (inter quartile range) [number of samples above detection limits of the method]. *p* values from Wilcoxon tests. Data summarized from *FASEB J* (2014) **28,** 4835-46 and *J Lipid Res.* (2016) 57, 1906-1916.

New terminology is called-for to highlight the latent rather than an inherent propensity of these mediators to elicit inflammation as an immunological response to injury. The generic term ‘*inflammins’* is coined to serve the purpose of emphasizing their dual role in inflammation and homeostasis. Thus, *unalamation* is a physiological process under homeostatic/developmental conditions mediated by lipid mediators such as PGE_2_, presumably under the functional balance from ‘anti-inflammatory’ lipid mediators [e.g. EpPUFA, generated by the cytochrome P450 metabolism of PUFA, endocannabinoids such as fattyacyl ethanolamides (45)] (**Figure 1**). The mediators that direct *inflammins* toward a homeostatic/developmental role are identified by the generic term ‘*unalamins’* rather than ‘anti-inflammatory’ mediators. Thus, both *inflammins* and *unalamins* are ‘homeostatic variables’, as defined by Medzhitov, whose functional quantitative balance makes the difference between homeostatic and inflammatory states (8). Such direct demonstration of *inflammin-unalamin* mediator balance in support of *unalamation* is hard to come by in normal healthy tissues due to practical and ethical considerations (even the transabdominal amniocentesis that helped the development of *unalamation* paradigm was indicated only for the assessment of potential genetic disorders). Blood is the most common biological sample for such biomarker measurements (we measure where we can rather than where we should). While concentration of the mediators can be high at the site of their biosynthesis, dilution of the effector mediators in systemic circulation minimizes their clinical significance when measured in peripheral circulation. Otherwise, COX-2 is constitutively expressed and results in abundant biosynthesis of PGE_2_ in tissues such as kidneys, intestine, lung, or brain, just to name a few, without clinical symptoms of inflammation (46, 47). Disruption of homeostasis in these tissues by non-steroidal anti-inflammatory drugs (e.g. gastrointestinal toxicity of NSAIDs) shall serve as evidence in support of the role of these ‘inflammatory’ mediators in *unalamation*.

As described above, the most obvious difference between acute and chronic inflammation is temporal extension of the clinical symptoms. While injury is the origin of acute inflammation, such etiological certainty is elusive for chronic inflammation. Is chronic inflammation a result of unresolved acute inflammation that manifests in temporal sub-clinical extension of symptoms? While literature is replete with conjectures of an extension of low intensity acute inflammation masquerading as chronic inflammation due to its incomplete resolution, cell biology suggests otherwise (**Table 2**). Acute inflammation is characterized by the adhesion of polymorphonuclear leukocytes to vascular endothelium followed by the opening of endothelial cell tight junctions resulting in extravasation. Clearance of the leukocytes following neutralization of the injurious agent leads to the reversal of the vascular leakage and initiates the resolution of acute inflammation. Adoptive immune surveillance mechanisms are predisposed to resolve acute inflammatory response to injury (13). Chronic inflammation on the other hand is morphologically defined by the presence of lymphocytes, macrophages, and plasma cells to activate fibroblasts in tissues to perpetuate inflammation (48). Despite the detection of lipid and protein mediators as *inflammins* under chronic inflammatory conditions, little is known about the status of *unalamins* in the same context. If we were to extrapolate the observations in sterile clinical chorioamnionitis to systemic chronic inflammatory diseases (e.g. rheumatoid arthritis, diabetes, neurodegenerative diseases, aging, etc.), a functional imbalance between *inflammins* and *unalamins* [perhaps driven by metabolic or genetic disorders (49)] offers a highly plausible biochemical explanation for the etiology of chronic inflammation.

**Table 2:** Contrasting acute and chronic inflammation.

	Acute	Chronic
Mediators	Lipids (e.g. PGE2) Proteins (e.g. cytokines)	Lipids (e.g. PGE2) Proteins (e.g. cytokines)
Cell Biology	Recruitment of Neutrophils (PMN) Endothelial cell activation Vascular leakage Platelet aggregation Fibrin deposition	Recruitment of Lymphocytes, Macrophages, Plasma cells and Giant cells. Granuloma formation Necrosis Collagen scar
Duration	Hours to days	Months to years
Etiology	Injury (microbial, exposure to foreign substances, physical damage)	Unknown (except for persistent sub-clinical exposure to injurious agents)
Resolution	Active	Unknown
Physiological Consequence	Defense	Disease
Therapeutic options	NSAIDs, Receptor antagonists	Immunosuppressive drugs and mediators of resolution of inflammation (SPMs)

## Defining chronic inflammation

**Figure 2:**
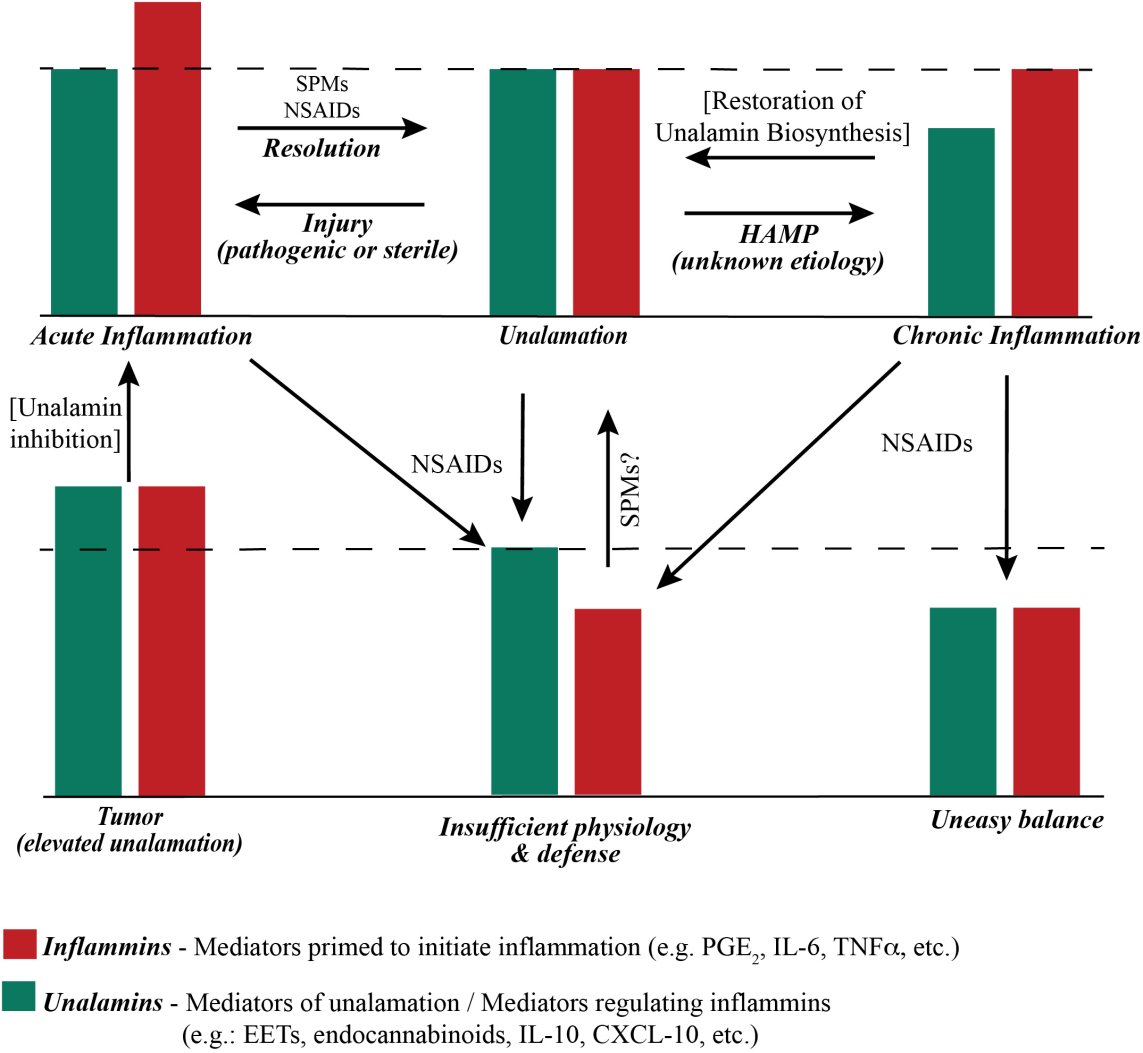
Functional stoichiometry of ‘inflammatory’ and ‘anti-inflammatory’ mediators. The dashed line represents the theoretical optimum *functional* (not actual concentration) balance between *inflammins* and *unalamins* for homeostasis. An *ad-hoc* increase in the function of *inflammins* from injury results in acute inflammation and immune response. Resolution of acute inflammation is mediated by pre-programmed biosynthesis of Specialized Pro-resolving Mediators (SPM) following adaptive immune response to injury. Resolution of acute inflammation is further assisted by Non-Steroidal Anti-Inflammatory Drugs (NSAIDs) that inhibit the biosynthesis of prostaglandins. A reduction in the functional level of *unalamins* also results in inflammation. If such reduction of *unalamins* is temporary, the result is still acute inflammation. However, a sustained lowering of the functional *unalamin* levels results in chronic inflammation. Either way it is a Homeostasis Altering Molecular Process (HAMP). NSAIDs can be employed to reduce the biosynthesis of *inflammins* in chronic inflammation to bring them in balance with *unalamins*. However, the system would be at sub-optimal level of *inflammin-unalamin* functional balance (i.e. below the theoretical optimum) and could reflect in sub-par physiological function for homeostasis. An ideal anti-inflammatory drug to treat chronic inflammation would be one that stimulates functional *unalamin* levels without affecting *inflammins*. It is possible that NSAIDs could also stimulate *unalamin* biosynthesis. However, limited success of NSAIDs in treating chronic inflammation suggests otherwise. Both *unalamins* and *inflammins* are present in tumor microenvironment at higher levels compared to normal tissues (55–57). This could lead to enhanced angiogenesis and cell proliferation, in other words, tumor growth. Targeted inhibition of *unalamin* biosynthesis in tumor tissue could lead to acute inflammation in the tumor microenvironment and trigger an immune response followed by resolution to restore homeostasis, i.e. ideal cancer therapy.

Acute inflammation because of infection (triggered by Pathogen Associated Molecular Patterns or PAMP) or physical injury (triggered by Damage Associated Molecular Patterns or DAMP) is a response of the adaptive immune system to restore homeostasis. Changes in cellular homeostasis outside of the ascertainable pattern recognition system was conceptually described as homeostasis altering molecular process (HAMP) (50). Disruption of *unalamation* resulting from a functional imbalance between *inflammins* and *unalamins* is an example of HAMP that could trigger inflammasome activation. Chronic inflammation would be the result of perturbation of homeostasis from a lowering of *unalamins* distorting *unalamation*. Hence, neutralization of PAMP or DAMP helps resolve the acute inflammation whereas only a restoration of *unalamin* biosynthesis reinstates *unalamation* and resolves chronic inflammation. This functional stoichiometry between *inflammins* and *unalamins* in acute and chronic inflammatory conditions is illustrated in **Figure 2**. From this model, it is imperative that traditional pharmacological approach of enzyme inhibition to reduce the biosynthesis or receptor antagonism to dampen the signaling of *inflammins* is less productive to treat chronic inflammation because of sub-optimal level of *unalamation*. Experience from the ineffectiveness of NSAIDs and anti-cytokine therapy in treating chronic inflammatory diseases attests to this shortcoming. Thus, *unalamation sustains homeostasis. Perturbed unalamation in the absence of injury results in chronic inflammation.*

## Implications of the etiology of chronic inflammation

Perturbation of the functional stoichiometry between *inflammins* and *unalamins* as the origin of chronic inflammation offers an appreciation of the difficulty in treating chronic diseases, not the least of which is cancer, with anti-inflammatory drugs. Notwithstanding the fact that anti-inflammatory drugs are not effective anticancer agents, chronic inflammation is viewed as an essential driver of tumor growth and progression (51–53). While tumor growth causes systemic imbalance, and hence detrimental to the organism, it is a proliferative disorder, not a destructive disease like neurodegenerative, musculoskeletal, cardiovascular, or other chronic diseases. Presence of ‘inflammatory’ mediators (e.g. PGE_2_, IL-6, etc.) in the tumor certainly supports the designation of an ‘inflammatory’ microenvironment (54). However, the tumor microenvironment is not at the exclusion of ‘anti-inflammatory’ mediators such as the EpPUFA and cytokines (55–57). It is not too farfetched to envision elevated levels of both the ‘inflammatory’ mediators such as PGE_2_ and the ‘anti-inflammatory’ mediators such as EpPUFAs working in concert to promote angiogenesis, cell proliferation, and evade immune surveillance. An elevated balance of *inflammins* and *unalamins* is a likely mechanism to promote and sustain tumor growth (37, 58). Thus, cancer, like any other normal tissue, is sustained by *unalamation* (but at a heightened state), not inflammation. This new paradigm is fully in concert with the updated hallmarks of cancer and provides a biochemical basis to the historical observations in cancer development and progression (59, 60).

This new conceptual understanding on the etiology of chronic inflammation suggests the following therapeutic predictions to treat chronic diseases as well as cancer:

Prediction 1: Degenerative chronic diseases can be treated by enhancing the biosynthesis of *unalamins* rather than inhibition of *inflammins* to return the system to optimal homeostasis. This can be achieved by the induction of Cytochrome P450-dependent epoxygenases. Alternatively, degradation of the EpPUFA by soluble epoxide hydrolase(s) could be inhibited, provided the degradative enzyme is active and the substrate (i.e. EpPUFA) is plentiful in these tissues (61). Given that nearly all pharmaceuticals are either enzyme inhibitors or receptor antagonists, this would be a new approach to drug design if the shortcoming is the biosynthesis of EpPUFA.

Prediction 2: Cancer therapeutics shall target not just the *inflammins* but *unalamins* as well to bring their balance to normal tissue level.

Prediction 3: Targeting anticancer therapeutics to inhibit the biosynthesis of *unalamins* to the level in normal tissues shall lead to functional balance in favor of *inflammins* in the tumor microenvironment. This can lead to immune surveillance of tumors by induction local acute inflammation. Inhibition of EpPUFA biosynthesis is known to induce the influx of monocytes giving credence to this strategy (37).

In conclusion, chronic inflammation is not an offshoot of acute inflammation. Etiology of chronic inflammation as a perturbation of *unalamation* offers effective strategies for the treatment of debilitating chronic diseases. Recognition of *unalamation* as a balance between the lipid mediators that can initiate inflammation and those that inhibit inflammation to sustain tissue homeostasis and development offers a fundamentally novel strategy for cancer treatment.

## Data Availability

Publicly available datasets were analyzed in this study. All data are in the published papers cited in the article.

## Footnotes

1 The name is derived from the Sanskrit word *anala* denoting mythical fire in stomach that helps sustain life.
